# Relationship between Body Mass Index and Physical Activity among Children from Low-Income Communities in Gqeberha, South Africa: A Cross-Sectional Study

**DOI:** 10.3390/ijerph20021428

**Published:** 2023-01-12

**Authors:** Danielle Dolley, Rosa Du Randt, Uwe Pühse, Markus Gerber, Jacob Bosma, Ann Aerts, Larissa Adams, Patricia Arnaiz, Nandi Joubert, Ivan Müller, Siphesihle Nqweniso, Harald Seelig, Peter Steinmann, Jürg Utzinger, Cheryl Walter

**Affiliations:** 1 Department of Human Movement Science, Nelson Mandela University, Gqeberha 77000, South Africa; 2 Department of Sport, Exercise and Health, University of Basel, 4052 Basel, Switzerland; 3 Novartis Foundation, 4056 Basel, Switzerland; 4 Swiss Tropical and Public Health Institute, 4123 Allschwil, Switzerland; 5 University of Basel, 4052 Basel, Switzerland

**Keywords:** children, body mass index, underweight, overweight, obesity, moderate-to-vigorous physical activity, low-income schools

## Abstract

This study aimed to establish the prevalence of underweight, overweight and obesity, the level of moderate-to-vigorous physical activity (MVPA) and the association thereof among vulnerable children from low-income communities in South Africa. Cross-sectional data were collected from 916 children (467 boys and 449 girls) aged 8–13 years ($\bar{x}$ = 10.4 ± 1.2 years) attending eight low-income schools in Gqeberha, South Africa. Measured outcomes included accelerometery-measured physical activity (PA), weight, height and body mass index (BMI). Analysis of variance was used to determine the mean difference of total MVPA stratified by sex and BMI classification. Overall, 13% of the cohort were underweight, 19% were overweight/obese and 64% engaged in 60 min of MVPA per day. Girls presented nearly twice the odds of being overweight or obese than boys (95% CI: 1.40–2.77). Underweight to normal-weight children (boys: OR = 3.89, 95% CI: 2.18–6.93; girls: OR = 1.78, 95% CI: 1.13–2.80) were more likely to engage in 60 min/day of MVPA than overweight to obese children. There is an inverse association between BMI categories and theduration of MVPA achieved per day. Special attention should be aimed at increasing awareness of healthy nutrition and promoting a variety of PA, especially among girls and children with excess weight.

## 1. Introduction

Non-communicable diseases (NCDs) are a leading cause of morbidity and account for seven of ten global mortalities [1]. Global efforts have emerged to prioritise the prevention and treatment of NCDs, which were included in the United Nation’s Sustainable Development Goals (target 3.4). Meanwhile, NCD mortalities are higher in low- and middle-income countries (LMICs) as access to primary healthcare needed to treat and manage NCDs is often lacking [1,2]. Moreover, while South Africa is classified as an upper-middle-income country, it is also ranked as the most unequal country in the world (0.63 Gini coefficient) [3], where social and economic inequalities exacerbate the NCD cycle.

South Africa has vastly unequal socioeconomic societies with diverse cultures, ethnicities and dietary preferences, which may impact the weight status of the different population groups [4]. Evidence describing national demographics have reported that the prevalence of underweight was highest amongst Coloured (mixed ancestry) children, whereas Caucasian children had the highest incidence of overweight and obesity compared to Black African and Coloured children [5]. Moreover, studies have found that children in more urbanised areas are more likely to suffer from overnutrition, while children from rural areas are mostly affected by stunting and being underweight [5,6]. Emerging research has also pointed to the role of maternal nutrition, as stunted mothers are more likely to have stunted and overweight infants who become obese children, thereby perpetuating the intergenerational cycle of malnutrition and diet-related NCDs [7,8].

South Africa is experiencing a nutritional transition, where the incidence of overweight and obesity continues to rise despite the high prevalence of micronutrient deficiencies and food insecurity. According to the South African Child Gauge Report, one in eight children are overweight or obese, and one in four are stunted [8]. These high proportions of malnutrition are consistent with poor dietary habits and a low intake of micronutrients [9]. A recent systematic review by Wrottesley, Pedro, Fall et al. [10] confirmed that South African children and adolescents are consuming higher quantities of energy-dense processed foods that are high in sugar and fat but low in micronutrients. A study (which assessed the food and nutrition environment of 16 low-income schools in the Eastern Cape province of South Africa) further corroborated these findings as it reported that a high incidence of unhealthy dietary practices was observed and that energy-dense food items such as fried bread dough (fat cakes), sweets and chips were the main items purchased at school tuckshops [11].

Unfortunately, children in LMICs are the most vulnerable and susceptible to nutritional imbalances as they may be exposed to inadequate nutrition during critical periods of their development [12]. A recent study investigated the shift in nutritional status in four selected African countries (South Africa, Ghana, Kenya and Malawi) with different socioeconomic development status. This study found a high prevalence of overweight and obesity (13%) compared to underweight (5.9%) among children under five years, although the incidence of stunting was considerably higher (27%) [13]. These rates are markedly higher than the global average. The 2018 Global Nutrition Report illustrated the extent of malnutrition, where every country in the world is afflicted by some form of malnutrition. It is reported that 150.8 million (22.2%) children (0–59 months) are stunted, and 38.3 million (5.6%) children are overweight [7].

There is an urgent need to address the excess consumption of refined grains and sugary food and beverages as this ‘Westernised diet’ negatively affects the health and wellbeing of children and increases the risk of NCDs in adulthood [14]. The consequences of such nutritional imbalances are likely to affect the individual’s quality of life and may impact the country’s health burden, productivity and growth potential. Unhealthy dietary habits and physical inactivity are established contributors to NCDs, which are associated with the risk of low self-esteem and psychiatric disorders during adolescence [15,16]. Thus, a strategy to curb intergenerational malnutrition and combat the rising prevalence of NCD risk is to promote healthy and active lifestyles from a young and impressionable age.

Physical activity (PA) is associated with numerous health benefits, which include the reduced risk of NCDs, improved body composition, self-perception and self-esteem and reductions in depression [17]. Evidence-based recommendations stipulate that children and adolescents should participate in at least 60 min of daily moderate-to-vigorous physical activity (MVPA) to realise these health benefits [18]. However, many children and youths are insufficiently active and do not meet these targets. The Global Matrix 4.0 confirms that the PA levels of children and youths are a critical public health concern. Estimates show that only one-third (27–33%) of children and adolescents meet the recommended amount of PA [19]. The recently published 2022 Healthy Active Kids South Africa (HAKSA) Report Card has shown more positive estimates, with 60% to 73% of children and adolescents achieving the recommended 60 min of MVPA/day [20]. Seemingly, the majority of South African children may be meeting the PA guideline, but vulnerable populations, in particular, girls, overweight and obese children, children with less-educated parents and those of a lower socioeconomic status (SES), are less likely to participate in the recommended amount of PA than boys, children of a normal-weight status, children with educated parents and those with a higher SES [21,22,23,24]. Evidence also shows that PA is likely to decrease with increasing age, with annual declines in MVPA starting from the ages of six years for girls and nine years for boys [25].

Against this background, the current study aimed to establish the prevalence of underweight, overweight and obesity as well as the level of MVPA among primary school-aged children from selected low-income communities in Gqeberha, South Africa. In addition, this study also sought to determine the association between children’s BMI classification and PA behaviour.

## 2. Materials and Methods

### 2.1. Study Design

The present study is a substudy of the larger *KaziBantu* project [26], which aims to improve the health conditions of both teachers and learners, thereby creating ‘Healthy Schools for Healthy Communities’ in under-resourced settings. The present study is a cross-sectional analysis of the baseline data collected for the *KaziBantu* study from January to March 2019 [26].

### 2.2. Participants

Eight quintile 3 primary schools from peri-urban areas of Gqeberha were included in the study. South African schools are ranked into five quintiles, from quintile 1, the poorest, to quintile 5, the least poor. Quintile 1–3 are non-fee-paying schools. Altogether, 1020 children from grades (Gr.) 4–6 (8–13 years, mean age: 10.4 ± 1.2 years) were recruited, and 975 were enrolled in the umbrella *KaziBantu* study. For the present study, 916 children (467 boys and 449 girls) had a complete data set for the body composition calculation.

### 2.3. Ethics Statements

Ethics approval was obtained from the Nelson Mandela University Research Ethics Committee (Human) (H19-HEA-HMS-003), the Eastern Cape Department of Health (EC_201804_007) and the Eastern Cape Department of Education. The study was registered with the ethical review board of the Ethics Committee Northwest and Central Switzerland (R-2018-00047). All required procedures were followed, including good clinical practice guidelines and the ethical principles defined in the Declaration of Helsinki [27]. The participant’s parents/guardians provided written informed consent, and each participant gave oral assent to participate in this study.

### 2.4. Anthropometric Measures

Body height was measured once to the nearest 0.1 cm using an SECA portable stadiometer as each child stood erect, shoulders relaxed and barefoot with their back and heels touching the height meter. Body weight was measured once to the nearest 0.1 kg using a wireless body composition monitor (Tanita MC-580) as each child stood barefoot on the scale with minimal clothing. Body composition was determined by calculating the BMI using the standard formula of weight (kg) divided by the square metre of height (m^2^). Underweight, normal, overweight and obesity status were assessed using age- and gender-specific cut-off points provided by the International Obesity Task Force (IOTF) and developed by Cole, Bellizzi, Flegal et al. [28].

### 2.5. Physical Activity

Device-based PA was measured using a light triaxial ActiGraph^®^ wGT3X-BT accelerometer (Actigraph LLC., Pensacola, USA), which has been proven to accurately measure the daily activity of children [29]. The device was fitted at the hip and worn for seven consecutive days, except during activities involving water contact. A 30 Hz sampling rate was used, and data were stored as GT3X raw files. Analyses were performed with the ActiLife software (version 6.13.2; Actigraph LLC, Pensacola, FL, USA) using 10-sec epoch lengths. Non-wear time was calculated and eliminated using the Troiano, Berrigan, Dodd et al. [30] algorithm. Evenson et al. (2008) cut points were used to calculate the PA intensities for children. The data were considered valid if the child wore the device for a minimum wear time, defined as total PA (6 am–12 am, ≥480 min/day on ≥4 weekdays and ≥1 weekend day) [31]. MVPA was calculated as minutes per day in moderate and vigorous intensities.

### 2.6. Statistical Analysis

The collected data were double-entered and validated in EpiData version 3.1 (EpiData Association; Odense, Denmark). All statistical analyses were obtained using Statistica^®^ version 13 (TIBCO Software Inc, Palo Alto, CA, USA) and Microsoft^®^ Office Excel 2016 (Microsoft Corporation, Redmond, WA, USA). Continuous variables were descriptively analysed by means (M) and standard deviations (SD). The various levels of the categorical variables were presented as percentages (%). The chi-squared test of independence was used to determine the differences in weight status in the compared groups. Analysis of variance was performed to determine the mean difference between total MVPA per sex and BMI classification. The level of statistical significance was set at *p* < 0.05. The effect sizes were determined using Cohen’s d, which are interpreted as follows: d < 0.2, no difference; d = 0.20–0.49, small difference; d = 0.50–0.79, medium difference; and d ≥ 0.80, large difference. Odds ratio (OR) analyses were also performed to determine the odds of children meeting ≥60 min MVPA/day depending on their BMI classification. A 95% confidence interval (CI) is presented for OR.

## 3. Results

Table 1 presents the descriptive statistics. The gender distribution of the sample is equally split (51% boys and 49% girls). The mean age of the boys was 3.3% higher than that of the girls; this difference showed a statistical significance (*p* < 0.005; d = 0.297). The mean height of the boys and girls in this sample was equal within the sampling error (*p* = 0.237; d = 0.078). The mean weight of the girls was 6.5% higher than that of the boys, and this difference was statistically significant (*p* < 0.001, d = 0.22). The mean BMI of the girls was 5.2% higher than that of the boys, and the difference was statistically significant (*p* < 0.001; d = 0.25). The average body fat % of the girls was almost 26% higher than that of the boys; this difference showed strong statistical significance (*p* < 0.001; d = 0.85). The mean total MVPA of the boys was 32.58% higher than that of the girls; this difference was statistically significant (*p* < 0.001; d = 0.93).

**Figure 1:**
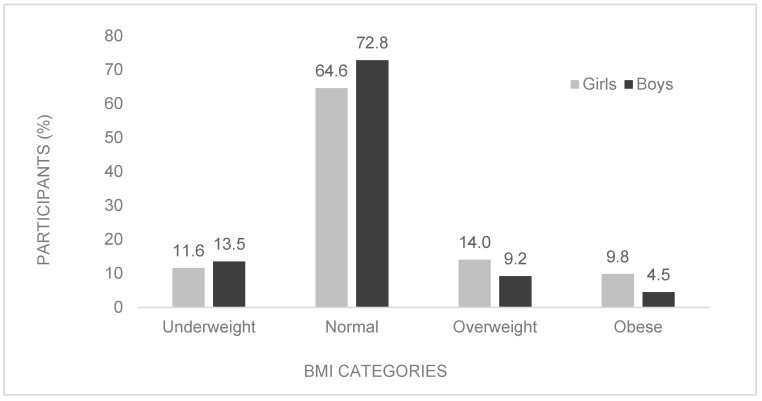
Prevalence of underweight, normal weight, overweight and obese, stratified by sex.

Figure 1 shows the results of the differences in weight status in the compared groups. Weight status was determined using the age- and sex-specific international cut-off points for the relevant BMI categories [29]. Most children were of normal weight (340 boys, 290 girls), while 13% (63 boys, 52 girls) were underweight, 12% were overweight (43 boys, 63 girls) and 7% were obese (21 boys, 44 girls). The chi-squared test revealed highly significant differences between the distribution of the BMI categories of the girls and boys (*p* < 0.001; Cramer’s V = 0.135). Cramer’s V interpretation is as follows: small = 0.06, medium = 0.17 and large = 0.29. The odds of being overweight or obese were nearly twice as high for girls (OR = 1.97, 95% CI: 1.40–2.77) than for boys.

**Figure 2:**
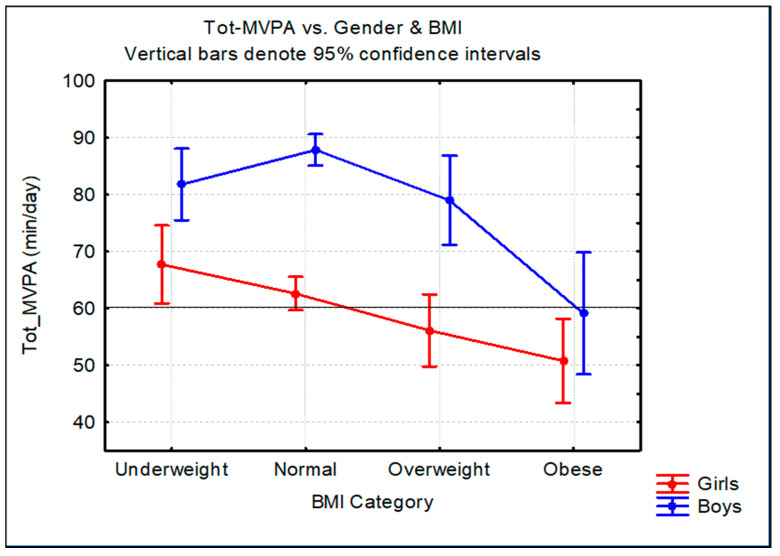
Mean total MVPA (min/day) stratified by sex and BMI categories.

Figure 2 shows the difference between the unweighted mean total MVPA in minutes/day according to sex and BMI categories. The ANOVA revealed significant differences. The mean total MVPA of the boys was significantly different from that of the girls for the underweight (boys: 81.8 min/day, 95% CI: 74.5–89.1 vs. girls: 67.7 min/day, 95% CI: 61.2–74.2), normal weight (boys: 87.8 min/day, 95% CI: 84.8–90.9 vs. girls: 62.6 min/day, 95% CI: 59.9–65.2) and overweight (boys: 79 min/day, 95% CI: 69.6–88.4 vs. girls: 56.1 min/day, 95% CI: 51.4–60.8) categories, respectively. However, the mean total MVPA of the obese boys did not differ significantly from that of the obese girls (boys: 59.1 min/day, 95% CI: 48.1–70.1 vs. girls: 50.8 min/day, 95% CI: 45.2–56.3).

**Figure 3:**
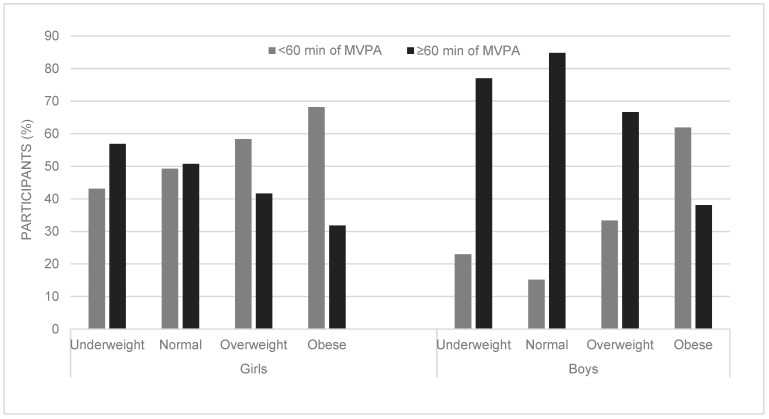
Percentage of children who did or did not meet ≥60 min MVPA, stratified by BMI categories and sex.

Figure 3 shows the percentage of underweight, normal-weight, overweight and obese children who did and did not meet the ≥60 min MVPA/day. The highest count of children who achieved ≥60 min MVPA/day was (in descending order) normal-weight (*n* = 410, 47%) and underweight (*n* = 76, 9%) children, followed by overweight (*n* = 51, 6%) and obese (*n* = 22, 3%) children.

A higher percentage of boys achieved ≥60 min of MVPA/day than girls in the underweight (47 boys, 77% vs. 29 girls, 57%; *p* = 0.029, Cramer’s V = 0.215), normal-weight (268 boys, 85% vs. 142 girls, 51%; *p* < 0.0001, Cramer’s V = 0.378) and overweight (26 boys, 67% vs. 25 girls, 42%; *p* = 0.021, Cramer’s V = 0.244) categories. In contrast, the difference between the percentages of obese boys and girls who achieved ≥60 min MVPA/day was not much different (8 boys, 38% vs. 14 girls, 32%; *p* = 0.352, Cramer’s V = 0.062). Cramer’s V interpretation is as follows: small = 0.1, medium = 0.3 and large = 0.5.

In the studied sample, obese girls were less likely to meet ≥60 min MVPA/day than overweight girls (OR = 1.53, 95% CI: 0.68–3.46). Moreover, the odds were much lower for obese boys to achieve ≥60 min of MVPA/day than for overweight boys (OR = 3.25, 95% CI: 1.08–9.80).

Table 2 presents the odds of being overweight or obese as a result of time spent in daily MVPA. Among the girls classified as overweight/obese, 62.5% (*n* = 65) engaged in <60 min of MVPA/day, while 37.5% (*n* = 39) engaged in ≥60 min of MVPA/day. The odds of engaging in ≥60 min/day are 1.78 times higher for underweight to normal-weight girls than overweight or obese girls (95% CI: 1.13–2.80, *p* = 0.017). Among the boys classified as overweight/obese, 43.3% (*n* = 26) engaged in <60 min of MVPA/day, whereas 56.7% (*n* = 34) participated in ≥60 min of MVPA/day. The odds of participating in ≥60 min/day are 3.89 times higher for underweight to normal-weight boys than overweight or obese boys (95% CI: 2.18–6.93, *p* < 0.0001).

## 4. Discussion

This study aimed to (1) establish the prevalence of underweight, overweight and obesity among 8–13-year-old children from selected quintile 3 (no-fee paying) schools in low-income peri-urban areas of Gqebeha in the Eastern Cape, South Africa and furthermore, (2) to establish whether children’s BMI was associated with their PA behaviour.

The BMI prevalence rates from the current cohort (*n* = 916, $\bar{x}$ age = 10.4 ± 1.2 years: 13% underweight, 12% overweight and 7% obese) fall within the prevalence rates of a systematic review of South African children and youths (0–20 years) [5], which reported values that ranged from 4% to 19% for underweight (using the National Centre for Health Statistics/WHO z-scores), 5.4% to 32.4% for overweight and 2.5% to 17.3% for obesity using the Cole-IOTF age- and gender-specific cut-off points [5,28].

Studies conducted in different geographic regions in South Africa have reported conflicting results on the prevalence of weight status among children and adolescents. A systematic review by Monyeki, Awotidebe, Strydom et al. [5] found a higher incidence of being underweight in rural areas. In contrast, a later review by Mbogori, Kimmel, Zhang et al. [13] reported that the prevalence of underweight and overweight/obesity was similar in rural and urban regions of South Africa, with a ratio of 1.0. Pretorius, Neophytou and Watson [6] also reported significant differences in undernutrition (16.1% vs. 9.5%, *p* < 0.001) and overnutrition (9.7% vs. 41.2%, *p* < 0.001) between the peri-urban areas of Gqeberha in the Eastern Cape and the urban areas of Johannesburg in Gauteng.

The BMI profiles of the present study were compared to the anthropometric profiles of the Gqeberha sample (*n* = 791, $\bar{x}$ age = 9.7 ± 0.8 years) measured by Pretorius and colleagues in 2016 [6]. Pretorius, Neophytou and Watson [6] reported a mean BMI of 17.5 ± 3.1 kg/m^2^, which was not much lower than the present study cohort’s BMI (18.0 ± 3.7 kg/m^2^) measured in 2019. Pretorius, Neophytou and Watson [6] reported similar prevalence rates for underweight (16.1%) and obesity (9.7%) compared to the current sample. However, the prevalence of overweight children (39.8%) was more than three times that of the current study, even though both cohorts were from peri-urban areas of Gqeberha and of similar SES.

Diet may explain the variations in weight status in different geographic areas. The study by Okeyo, Seekoe, de Villiers et al. [11] in the Eastern Cape found that compared to urban areas, the frequency of eating breakfast, sugary snacks and drinking cordial (a sweetened concentrate mixed with water) was higher among learners in rural areas, whereas those in urban areas consumed more boerewors (beef sausage), hamburgers and sugar-sweetened beverages. Wrottesley, Pedro, Fall et al. [10] found that factors such as irregular breakfast consumption, fewer meals with families and increased snacking were associated with increased weight and obesity among adolescents. It is also plausible that the differences in weight status could be due to different BMI reference charts used, which may underestimate or overestimate the incidence of underweight, overweight and obesity [5]. However, other factors such as ethnicity or culture could also play a role [4].

Adolescence is a life stage of significant anatomical and physiological changes with increased nutrient needs [7]. It is also a period where children and youths are highly susceptible to adopting unhealthy habits, which increase their risk of future NCDs. In the studied sample, the odds of being overweight or obese were nearly twice as high for girls (OR = 1.97, 95% CI: 1.40–2.77) than for boys (Figure 1). A plausible explanation is diet, since Okeyo, Seekoe, de Villiers et al. [11] found gender-related differences in eating habits as girls ate significantly more processed and sugary foods (hot dogs, fat cakes, candy and chips) than boys. The literature also suggests that gender-related discrepancies in weight status are likely due to differences in energy needs, the onset of sexual maturity and levels of PA [32]. Results from this study also showed that the girls (61.01 min of MVPA/day, 95% CI: 58.99–63.03) were significantly less active than boys (84.76 min of MVPA/day, 82.14–87.39) (Table 1). An Australian study conducted by Telford, Telford, Olive et al. [22] found that low PA levels among girls were associated with weak school, community and parental support, suggesting fewer opportunities for PA or less support for PA participation among girls. Telford, Telford, Olive et al. [22] also observed less favourable attributes (lower perceived competence in physical education (PE), lower cardiorespiratory fitness and higher body fat percentage) associated with PA among girls. Another study, conducted in the Western Cape (South Africa) among adolescents, found that programmes which do not meet the needs and interests of participants can severely discourage PA participation [33]. Other barriers reported to hinder PA participation among adolescent girls in South Africa include weather, safety, time constraints and fears of weight loss instead of having a fuller figure [10].

According to the new WHO 2020 guidelines on PA, “average of” 60 min MVPA is recommended for children and youths (5–17 years) [18]. Overall, 64% (*n* = 559) of the studied sample engaged in ≥60 min of total MVPA/day, of which more than half (62%) were boys. Findings from this study show that overweight to obese children were less likely to meet the 60 min of MVPA per day than underweight to normal-weight children (boys: OR = 3.89, 95% CI: 2.18–6.93; girls: OR = 1.78, 95% CI: 1.13–2.80) (Table 2). Moreover, obese children were less likely to meet ≥60 min MVPA/day than overweight children (Figure 3). Obese girls were 1.53 times less likely to meet ≥60 min MVPA/day than overweight girls (95% CI: 0.68–3.46), but obese boys were of particular concern as the odds (OR: 3.25 times, 95% CI: 1.08–9.80) were much lower for obese boys to meet the ≥60 min of MVPA/day than overweight boys. Our findings correlate with the literature, as research shows that children who are overweight or obese are typically less active as they display poorer physical competence as well as lower perceived physical competence [15]. PA’s physical and psychological benefits are realised at an average of 60 min of MVPA per day [17,18]. Therefore, overweight and obese children should be encouraged to be more active and taught the value of a healthy and active lifestyle since regular MVPA is pertinent to correcting their body weight and modifying their metabolic disease risk [5].

### Strengths and Limitations

Despite efforts, our study did have limitations. Previous studies have investigated body composition in relation to different ethnic groups; however, this was not the purpose of the current study, which only concentrated on sex comparisons among children attending low-income schools. The cross-sectional design of this study precludes inferences of causal associations. Furthermore, the study findings can not be generalised to all South African children as it was conducted in one metropolitan area in the Eastern Cape province (one of the nine South African provinces). Strengths of the study include a relatively large study sample focusing on a vulnerable community. The results are also based on the true measurements of height, weight and device-measured PA via accelerometers. An added strength of this study is quantifying the odds of children meeting the MVPA recommendations in relation to their BMI classifications. The findings from this study, a substudy of the larger *KaziBantu* project, also provide evidence of what may be required to achieve a ‘Healthy Schools for Healthy Communities’ environment in under-resourced settings.

## 5. Conclusions

We found a concerning incidence of underweight, overweight and obesity in the studied cohort of primary school children attending low-income schools in Gqeberha, South Africa. The profiling of malnutrition and physical inactivity is essential to determine disease risk and to define appropriate interventions. Findings showed that girls and overweight to obese children were less likely to be active than boys and underweight to normal-weight children. Furthermore, obese boys in particular were less likely to achieve the average 60 min MVPA/day than overweight boys. Girls were also identified as a vulnerable group requiring special attention in promoting and adhering to PA. PE at schools is crucial to children’s all-round wellbeing and plays a vital role in developing an active lifestyle. PE educators are therefore encouraged to deliver equally engaging lessons for girls and children with excess weight to expose them to different modes of PA they may enjoy. In addition, the Department of Basic Education’s role in this regard through the in-service training of PE teachers and other support is also recommended, especially within marginalised schools and communities. Promoting healthy and active lifestyles is important to curb future disease risk. Thus, future research intervention strategies addressing physical inactivity in South Africa should focus on sub-groups, such as girls and children with excess weight. Concerted efforts should also be made to inform children, parents and teachers alike about the role of PA and healthy eating as a preventative strategy to mitigate future NCD risk. Accompanying longitudinal research may also investigate the personal and social supportive roles of family members, peers and teachers for PA.

## Figures and Tables

**Table 1:** Descriptive statistics were presented for the whole group and by sex comparison.

	Total (*n* = 975)		Boys (*n* = 474)		Girls (*n* = 501)			
	*n*	$\bar{x}$ (SD)	*n*	$\bar{x}$ (SD)	*n*	$\bar{x}$ (SD)	*p*-Value	Cohen’s d
Age (years)	916	10.4 (1.2)	467	10.6 (1.2)	449	10.2 (1.1)	<0.001 *	0.297
Height (cm)	927	139.8 (8.8)	473	139.5 (8.7)	454	140.2 (9.0)	0.237	−0.078
Weight (kg)	917	35.6 (10.1)	467	34.5 (9.1)	450	36.7 (11.0)	0.001 *	−0.221
BMI (kg/m^2^)	917	18.0 (3.7)	467	17.5 (3.2)	450	18.4 (4.0)	<0.001 *	−0.250
Body fat (%)	922	23.64 (6.88)	471	21.00 (6.37)	451	26.41 (6.28)	<0.001 *	0.85
MVPA (min/day)	920	72.99 (28.25)	464	84.76 (28.80)	456	61.01 (21.96)	<0.001 *	−0.93

Notes. Statistical significance at * *p* < 0.05. The variation in the *n* values per variable results from missing data. The effect sizes (Cohen’s d) are interpreted as: d < 0.2, no difference; d = 0.20–0.49, small difference; d = 0.50–0.79, medium difference; d ≥ 0.80, large difference. $\bar{x}$ = Mean; SD = standard deviation, BMI = body mass index.

**Table 2:** Prevalence of meeting MVPA criteria and the relevant odds ratios for girls and boys, respectively.

	Daily MVPA							
	<60 min		≥60 min			95% CI		
Sex	*n*	%	*n*	%	OR	LL	UL	*p*-value
Ov/Ob Girls	65	62.5	39	37.5	1.78	1.13	2.80	0.017 *
Ov/Ob Boys	26	43.3	34	56.7	3.89	2.18	6.93	<0.0001 *

Notes: Ov = Overweight. Ob = Obese. MVPA = Moderate-to-vigorous physical activity. CI = Confidence interval. OR = Odds ratio. LL = Lower limit. UP = Upper limit. Statistical significance at * *p* < 0.05.

## Data Availability

The data sets supporting the conclusions of this article are available from the corresponding author upon request.
